# Health and Social Problems Associated with Recent Novel Psychoactive Substance (NPS) Use Amongst Marginalised, Nightlife and Online Users in Six European Countries

**DOI:** 10.1007/s11469-017-9824-1

**Published:** 2017-12-07

**Authors:** Marie Claire Van Hout, Annemieke Benschop, Michal Bujalski, Katarzyna Dąbrowska, Zsolt Demetrovics, Katalin Felvinczi, Evelyn Hearne, Susana Henriques, Zsuzsa Kaló, Gerrit Kamphausen, Dirk Korf, Joana Paula Silva, Łukasz Wieczorek, Bernd Werse

**Affiliations:** 1 Public Health Institute, Liverpool John Moores University, Liverpool, UK; 2 Bonger Institute, University of Amsterdam, Amsterdam, Netherlands; 3 Institute of Psychiatry and Neurology, Warsaw, Poland; 4 Institute of Psychology, Eötvös Loránd University, Budapest, Hungary; 5 School of Health Sciences, Waterford Institute of Technology, Waterford, Ireland; 6 Centre for Research and Studies in Sociology, University Institute of Lisbon, Lisbon, Portugal; 7 Goethe-Universität, Frankfurt, Germany

**Keywords:** Novel psychoactive substance, NPS, Marginalised drug user, Online drug user, Nightlife, Health and social consequences

## Abstract

Continued diversification and use of new psychoactive substances (NPS) across Europe remains a public health challenge. The study describes health and social consequences of recent NPS use as reported in a survey of marginalised, nightlife and online NPS users in the Netherlands, Hungary, Portugal, Ireland, Germany and Poland (*n* = 3023). Some respondents were unable to categorise NPS they had used. Use of ‘*herbal blends*’ and ‘*synthetic cannabinoids obtained pure*’ was most reported in Germany, Poland and Hungary, and use of ‘*branded stimulants*’ and ‘*stimulants/empathogens/nootropics obtained pure*’ was most reported in the Netherlands. Increased heart rate and palpitation, dizziness, anxiety, horror trips and headaches were most commonly reported acute side effects. Marginalised users reported substantially more acute side effects, more mid- and long-term mental and physical problems, and more social problems. Development of country-specific NPS awareness raising initiatives, health and social service needs assessments, and targeted responses are warranted.

The continued diffusion, diversification and use of new psychoactive substances (NPS) across Europe remains a public health, drug policy and legal challenge (Arfken et al. 2014; Reuter and Pardo 2017), with science, surveillance and law enforcement struggling to keep up with the increasing range of new designer NPS marketed to circumvent legislative controls (Hillebrand et al. 2010; Schmidt et al. 2010; Gunderson et al. 2012; Caudevilla et al. 2013; United Nations Office on Drugs and Crime, UNODC 2013; Seely et al. 2012; Spaderna et al. 2013; Corazza et al. 2013; Brents and Prather 2014; Papaseit et al. 2014; EMCDDA 2015; Caudevilla 2016; Van Hout and Hearne 2017; Kassai et al. 2017). The UNODC and the European Union (EU) define NPS as ‘Substances of abuse, *either in a pure form or a preparation, that are not controlled by the 1961 Single Convention on Narcotic Drugs or the 1971 Convention on Psychotropic Substances, but which may pose a public health threat*’ (UNODC 2013; Council of the European Union decision 2005/387/JHA 2005; Martinotti et al. 2015, 295; EMCDDA 2016). In general, NPS represent a multitude of synthetic and natural compounds marketed as legal alternative to conventional illicit drugs, the most popular being cathinone derivatives, other amphetamine-type substances, and synthetic cannabinoids, observed since 2007 (Caudevilla 2016; EMCDDA 2016). By 2015, EMCDDA identified 101 NPS, in addition to the 450 NPS already monitored in their surveillance systems (EMCDDA 2015). Seven categories of NPS are adopted in the scientific literature, namely the stimulant/cathinone; GABA activating; hallucinogen, dissociative, cannabinoid, opioid and other/unspecified/uncategorised NPS (Van Hout and Hearne 2017). Sourcing remains an issue with ongoing availability online and offline interactions via friends and local contacts (Lavorgna 2014, 2016; Sande 2016; Soussan and Kjellgren 2016; van Amsterdam et al. 2015; Van Hout and Hearne 2017), despite legislative controls in street, nightlife and online markets (McElrath and O’Neill 2011; Wood et al. 2012; EMCDDA 2015; Caudevilla 2016; Van Hout and Hearne 2017; Reuter and Pardo 2017).

Eurobarometer surveys report that 8% of young Europeans have experience of use of NPS (Eurobarometer 2014). Typically, NPS users are young males (Werse and Morgenstern 2012), with several focuses regarding groups of users: These include more mature and experienced ‘*psychonauts*’ operating on the Surface Web and Dark Net (Soussan and Kjellgren 2014; Van Hout and Bingham 2013a, b; Van Hout and Hearne 2017). In some countries and/or regions, NPS use also occurs amongst early adopter drug scenes, for example gay and club dance scenes, and student populations (Psychonaut Web Mapping Research Group 2009; Europol-EMCDDA 2010; Dargan et al. 2010; Carhart-Harris et al. 2011; Measham et al. 2011a; Johnston et al. 2015) and street drug users who inject (Pas 2010; Colfax et al. 2010; Europol-EMCDDA 2010; Brauser 2012; Van Hout and Bingham 2012; Csák et al. 2013; Van Hout 2014a; Kapitány-Fövény et al. 2015; Hearne et al. 2016; Rácz et al. 2016). Motivations to use NPS are similar to conventional illicit drug use motives and include curiosity, drug and sexual pleasure, sensation seeking, self-exploration, street, peer and online availability, perceptions around value for money and legality, poor quality of available conventional illicit drugs, preferred desired and duration of effects, and habit or dependent use (Werse and Morgenstern 2012); Bourne et al. 2014; Soussan and Kjellgren 2016; Van Hout 2014b; van Amsterdam et al. 2015; Van Hout and Hearne 2015; Global Drug Survey (GDS) 2016; Schmidt et al. 2016). The engagement of NPS users and interested parties within online drug fora for information sharing and advice around harm reduction is also increasingly observed (Hearne and Van Hout 2016; Rosino and Linders 2015; Soussan and Kjellgren 2015, 2016; Van Hout 2014a, b; Van Buskirk et al. 2016; Van Hout and Hearne 2015, 2017).

Social and legal consequences generally include the effects of NPS use on individuals, families, communities and the society in general. Use of NPS is associated with a range of acute and chronic health harms which are dependent on user characteristics and vulnerabilities, environmental characteristics, level of dosage, toxicity, route of administration and the presence of other substances. Such harms generally centre on adverse cardiovascular, respiratory and gastro-intestinal consequences of use, transmission of blood borne viruses (HIV, Hepatitis C), neurological and psychiatric harms (for example psychosis and suicidal ideation), dependence and death (Every-Palmer 2010; Hadlock et al. 2011; Maan and D’Souza 2011; Lusthof et al. 2011; Werse and Morgenstern 2012; Kasick et al. 2012; Grandy 2012; Seely et al. 2012; Gunderson et al. 2012; King and Nutt 2014; Abdulrahim et al. 2015; Van Hout 2016).

We report here on the declared acute and chronic health consequences of NPS use, and incurred social and legal problems as reported by the three groups of identified European NPS users, namely, *socially marginalised users* who are high risk drug users often unemployed, homeless and/or in care; *nightlife users* who are recreational drug users frequenting clubs, raves and/or festivals often in education and/or employed; and *users active on the Internet* and carrying out their NPS activity in online communities known as drug fora. The aim of the study was to describe health, social and legal consequences of NPS use as reported by NPS users resident in these six European countries. The research questions centred on investigating if there are associations of certain problems with groups of NPS users; whether there are differences between countries within these groups; and what associations occur in current use of certain categories of NPS.

## Methods

This study was undertaken within the remit of a transnational and interdisciplinary EU funded research project undertaken by researchers from different scientific disciplines in six EU-countries: Germany, Hungary, Ireland, the Netherlands, Poland and Portugal. A survey was designed based on extensive literature reviewing, country reports and expert consultations in each country, and which investigated user demographic characteristics, prevalence and patterns of use of illicit drugs; prevalence and patterns of NPS use; and reported health and social problems associated with use of NPS. Information was gathered on the use of seven categories of NPS: ‘*herbal blends* (e.g. “*Spice*”)’; ‘*synthetic cannabinoids (obtained pure)*’; ‘*branded stimulants* (e.g. “*bath salts*”)’; ‘*stimulants/empathogens/nootropics* (*obtained pure*, *e.g. mephedrone*, *MDPV*, *a-PVP*)’; ‘*psychedelics* (e.g. *NBOMe-x, 2C-x*’); ‘*dissociatives* (e.g. *methoxetamine*)’; and ‘*other*’. The questionnaire was developed in English and translated into the other languages in this project. To guarantee comparability, translation was done back and forth (from/into English).

Fieldwork occurred in the first 10 months of 2016. Eligibility criteria for the survey were (1) recent NPS use (at least once in the past 12 months), (2) resident of the participating countries and (3) 18 years or older. A targeted sampling methodology was chosen to reach NPS users in each of the three groups in the six participating countries:

1. Socially marginalised users were recruited face-to-face in the street, at or through user organisations and care facilities (e.g. low threshold facilities, consumption rooms) and through snowball sampling. Respondents were asked to either self-complete a pen-and-paper questionnaire, have a fieldworker read out the questions and record their answers, or complete an online questionnaire to which they were referred by a flyer containing a link and an individual code. In most cases, pen-and-paper questionnaires were interviewer-administered.
2. Users in night life were recruited face-to-face on-site at clubs, raves and festivals and through snowball sampling. Respondents were given the choice to self-complete either the pen-and-paper or online questionnaire.
3. Users in online communities were recruited by actively promoting the survey on internet fora and other online resources. Users in online communities were only given access to the online questionnaire.

The data was screened/cross-checked for errors and analysed using SPSS V.24. Analysis was conducted using anonymised data and involved descriptive statistics, including frequencies and percentages. Statistical tests included chi-square tests to assess differences in categorical data as well as ANOVA for mean comparison tests. Multivariate binary regression analyses were performed to determine which factors were associated with acute unpleasant side effects, mid- or long-term problems and social problems. In all statistical analyses, a two-sided significance level of 5% was used.

Respondents were asked about their use of NPS as it related to the seven categories; ‘*herbal blends*’; ‘*synthetic cannabinoids obtained pure*’; ‘*branded stimulants*’; ‘*stimulants/empathogens/nootropics obtained pure*’; ‘*psychedelics*’; ‘*dissociatives*’ and ‘*other*’. The survey responses revealed that some respondents were unable to categorise NPS they had used. This was especially evident in the categories of ‘*herbal blends*’ and ‘*synthetic cannabinoids obtained pure*’, and ‘*branded stimulants*’ and ‘*stimulants/empathogens/nootropics obtained pure*’*.* Names of NPS were not always entered under their proper categories; respondents were unaware of active ingredients and names of NPS they had used. For this reason, for reporting purposes, these categories were combined.

## Results

Three thousand five hundred three respondents were recruited, with 260 excluded as per eligibility criteria (residency/below 18 years). Of the remaining 3243 respondents, 212 were excluded because they either reported not using any NPS in the past 12 months (189/212), they answered or obviously answered the detailed questions on prevalence for non-NPS substances only, or answered negatively to the specific NPS categories. Eight further respondents were removed due to angry, incoherent or irrelevant responses in the open-ended text boxes.

## Respondent Profile

The final data set for analysis was 3023 recent NPS users. See Table 1. The online community sample was the largest (2110) and the marginalised sample was the smallest (266) across the six countries. Recruitment complexities such as low reporting of recent NPS use among certain categories of drug users in different countries was particularly evident in the marginalised respondents from the Netherlands and Portugal, and night life respondents from Ireland, with less than 10 respondents.

**Table 1:** Sample countries

	Marginalised	Night life	Online	Total
Sample				
Germany	23	98	542	663
Hungary	101	15	156	272
Ireland	48	3	11	62
The Netherlands	1	189	1000	1190
Poland	86	172	338	596
Portugal	7	170	63	240
Total	266	647	2110	3023

In terms of examining *all three samples across the six countries*, more than three thirds of respondents were male (marginalised 71.7%, night life 67.9%, online 68.0%). See Table 2. The online community respondents were youngest with an average age of 23.6 years, with 70.3% falling within the 18–24 age group. The night life respondents were on average 25.7 years. The marginalised respondents were oldest across all countries with an average of 33.5 years. In terms of location, the majority of marginalised and night life respondents were resident in large towns with more than 100.000 inhabitants (86.8/68.5%, respectively). Most of the night life and online community respondents lived either with their parents or family (27.6 and 44.1%), or in a rented accommodation (47.1 and 39.6%). A substantial proportion of the marginalised respondents in contrast reported living in homeless accommodations or hostels (32.3%) or other living arrangements (12.3%), including living on the streets (sleeping rough). Both night life and online respondents reported higher levels of education. Many had completed a secondary level education (46.2/56.2%, respectively), were studying for a higher degree (28.6/43.7%, respectively), or reported having a college or university diploma (37.3/28.6%, respectively). The majority of the marginalised respondents had completed none or just primary education (55.2%), with a minority being students (3.1%). The majority also were unemployed and/or living on benefits (75.7%).

**Table 2:** Respondent profile

	Marginalised *n* = 266	Night life *n* = 647	Online *n* = 2.110	*p*
Gender				
Male	71.7%	67.9%	68.0%	.457
Female	28.3%	21.1%	32.0%	
Age				
18–24 years	17.4%	50.1%	70.3%	.000
25–34 years	36.7%	41.7%	23.7%	
35–44 years	35.9%	7.6%	5.1%	
45 years and older	10.0%	0.6%	1.0%	
Average (sd)	33.5 (8.7)	25.7 (5.9)	23.6 (5.8)	.000
Residence				
Small town (pop. < 50.000)	6.1%	17.3%	41.2%	.000
Medium town (pop. 50.000–100.000)	8.1%	14.0%	17.0%	
Large town (pop. > 100.000)	85.8%	68.6%	41.8%	
Living arrangements				
Own home	6.9%	20.8%	14.5%	.000
Rent apartment or room	14.6%	47.1%	39.6%	
Parents/family	16.5%	27.6%	44.1%	
Friend’s home	9.6%	3.4%	1.1%	
Residential care	7.7%	0.6%	0.2%	
Homeless accommodation/hostel	32.3%	0.3%	0.2%	
Other	12.3%	0.2%	0.4%	
Level of education				
None	7.7%	0.3%	0.6%	.000
Primary school	47.5%	16.9%	12.8%	
Secondary school	37.9%	45.2%	56.2%	
College/university	5.7%	37.3%	28.5%	
Doctor’s degree, PhD, etc.	1.1%	0.3%	1.9%	
Employment				
Student	3.1%	28.6%	43.7%	.000
Full-time worker	3.5%	39.0%	30.6%	
Part-time or casual worker	9.4%	12.0%	11.7%	
Self-employed	5.9%	9.3%	5.4%	
Unemployed/benefits	75.7%	10.8%	8.1%	
Other	2.4%	0.3%	0.5%	

## Use of NPS

In terms of *categories of NPS* used, when comparing the six countries, use of ‘*herbal blends*’ and ‘*synthetic cannabinoids obtained pure*’ was most reported in Germany, Poland and Hungary, and use of ‘*branded stimulants*’ and ‘*stimulants/empathogens/nootropics obtained pure*’ was most reported in the Netherlands. See Table 3. Recent use of ‘*psychedelics*’ was most commonly reported by respondents from Portugal and Germany. To some degree, these results reflect the different sizes of subsamples in different countries, e.g. with Portuguese respondents mainly coming from nightlife settings, or Hungarian respondents who often were marginalised users.

**Table 3:** Life time use of NPS categories

	Herbal blends and/or pure synthetic cannabinoids		Branded and/or pure stimulants		Psychedelics		Dissociatives		Other NPS	
Sample										
Marginalised	73.3%	.000	85.0%	.000	22.2%	.000	22.6%	.896	10.9%	.000
Night life	55.8%		73.1%		56.7%		21.6%		13.0%	
Online community	46.2%		79.9%		51.3%		21.3%		34.3%	
Country										
Germany	63.0%	.000	50.1%	.000	75.9%	.000	33.9%	.000	37.6%	.000
Hungary	89.7%		79.4%		26.1%		28.3%		32.7%	
Ireland	48.4%		88.7%		21.0%		11.3%		17.7%	
The Netherlands	16.6%		96.7%		40.0%		10.3%		21.8%	
Poland	81.5%		82.9%		40.3%		27.9%		30.4%	
Portugal	64.2%		57.1%		85.8%		21.7%		19.6%	

When looking at frequent use of particular NPS categories, marginalised users were much more likely to show such patterns than the other two groups: 17.9% of the marginalised respondents reported daily herbal blend/synthetic cannabinoid use, compared to 1.2% of the night life and 2.8% of the online respondents. The respective figures for daily stimulant NPS use are 18.2% (marginalised), 0.3% (night life) and 1.0% (online). In terms of route of administration, the majority of marginalised respondents reported life time intravenous use (74.4%), compared to 3.6% of night life and 4.9% of online respondents. These observations were also reflected in the modes of NPS administration: while 51.1% of the marginalised users mentioned injection as the preferred mode of administration, only 0.8% of the other groups did so. Overall, swallowing was the most prevalent mode (online 61.6, night life 45.6, marginalised 2.6%).

## Side Effects of NPS Use and Associated Health and Social Problems

Within all three subsamples, the majority of recent NPS users had experienced acute unpleasant side effects after NPS use. See Table 4. In terms of reporting of acute side effects, experiences of increased heart rate and palpitation, dizziness, anxiety and horror trips and headaches were reported as most common across all three categories. The proportion who had experienced these effects was substantially larger in the marginalised sample (85.3%), than in the night life and online community samples (58.8 and 51.0%). When looking at the separate side effects, there are significant differences between the three groups in every symptom, with mostly much higher proportions of marginalised users reporting these effects. When comparing night life users and online community users, night life users reported more unpleasant effects, especially head and stomach aches and dizziness. However, in all categories, marginalised users show much higher rates than the two other groups. Increased heart rate or palpitation was the most reported side effect in all three samples.

**Table 4:** Side effects and problems from NPS use among marginalised, night life and online NPS users

	Marginalised *n* = 266	Night life *n* = 647	Online *n* = 2.110	*p*
Acute unpleasant side effects				
Increased heart rate/palpitation	57.5%	37.7%	35.8%	.000
Headaches	51.5%	26.6%	18.9%	.000
Stomach ache	38.0%	18.2%	12.8%	.000
Dizziness	44.7%	29.2%	20.7%	.000
Muscle cramps	33.1%	15.0%	12.7%	.000
Loss of consciousness/coma	23.3%	9.9%	6.7%	.000
Anxiety/horror trips	47.7%	25.5%	21.2%	.000
Shortness of breath	36.8%	16.0%	13.1%	.000
Hyperthermia	33.5%	17.8%	17.7%	.000
Aggression/violence	35.7%	11.1%	4.9%	.000
Paranoia	52.3%	21.9%	17.7%	.000
Other	4.1%	4.9%	6.3%	.221
Total	85.3%	58.8%	51.0%	.000
Mid- or long-term problems				
Addiction/withdrawal/craving	46.2%	7.4%	12.1%	.000
Depression	43.2%	13.6%	14.5%	.000
Paranoid disorders	38.7%	9.6%	8.2%	.000
Weight loss	46.2%	9.6%	9.5%	.000
Harms of injecting	26.7%	0.5%	0.8%	.000
Other mental problems	15.8%	6.3%	8.6%	.000
Other physical problems	12.4%	4.2%	6.4%	.000
Total	70.3%	28.4%	30.9%	.000
Social problems				
Conflict at school, university, etc.	6.8%	4.3%	4.7%	.277
Conflict at work	12.0%	5.6%	3.8%	.000
Conflicts with partner or family	45.9%	13.6%	12.8%	.000
Problems regarding housing	42.5%	3.7%	3.1%	.000
Legal problems	35.7%	6.2%	5.5%	.000
Other	3.0%	1.1%	1.7%	.117
Total	69.1%	22.1%	22.2%	.000

Marginalised users when compared to the other two categories reported also more mid- and long-term mental and physical problems (addiction withdrawals and craving, weight loss, depression and paranoid disorders) (70.3%) than the night life and online community samples (28.4 and 30.9%). In terms of mid- to long-term consequences of use, addiction withdrawals and craving were reported by almost half of marginalised users along with weight loss, depression and paranoid disorders. In comparison, such experiences were reported by a minority of online and nightlife users. Across all three categories, however, paranoid disorders, depression, weight loss and symptoms of addiction were most common. Marginalised users also reported more social problems as a consequence of NPS use than the other groups of users (mainly conflicts with partner or family, problems with housing and legal problems) (69.1 versus 22.1 and 22.2%). Low proportions of online and nightlife users reported social or legal problems. If any, respondents from these groups were most likely to report conflicts with partner or family, while, in contrast to the marginalised users, problems regarding housing and legal problems were relevant for very few nightlife and online respondents. See Table 4.

The proportions of different problems experienced by the respondents vary substantially by country of residence. See Table 5. Irish, Hungarian and Polish respondents were generally much more likely to report problems than Dutch or Portuguese users, with German users ranking in between. When looking at different types of problems, mid- and long-term problems, including social problems, were most prevalent in Ireland and Hungary. However, to a substantial degree, these differences reflect the varying distribution of user groups in the national samples (see Table 1 and the later ‘regression analysis’). When looking at side effects and problems among those who have experience in the use of different NPS categories, some differences were evident. See Table 5; no comparative statistical analysis given here because of overlapping groups. Overall, these results show a consistent picture that dissociatives and synthetic cannabinoids are the substances that are mostly associated to such problems. Interestingly, this is true for nearly all kinds of problems, no matter if physical, mental or social, and no matter if these are long-term (including addiction and withdrawal) or acute effects.

**Table 5:** Side effects and problems from NPS use per country and per lifetime use

	Acute unpleasant side effects		Mid- or long-term problems		Social problems	
Country						
Germany	62.2%	.000	26.9%	.000	22.9%	.000
Hungary	78.7%		63.7%		60.4%	
Ireland	95.2%		70.5%		74.6%	
The Netherlands	35.7%		24.7%		9.5%	
Poland	78.5%		49.3%		47.2%	
Portugal	42.2%		15.5%		15.2%	
Life time use						
Herbal blends and/or pure synthetic cannabinoids	69.7%		42.6%		38.6%	
Branded and/or pure stimulants	56.4%		37.2%		28.8%	
Psychedelics	56.8%		30.9%		25.6%	
Dissociatives	72.1%		44.5%		39.8%	
Other NPS	61.0%		39.4%		34.4%	

## Regression Analyses

Results show that negative experiences from NPS use are related to both type of user, country and categories of NPS used. Multivariate binary regression analyses was conducted to determine which of these three factors was most strongly associated with acute unpleasant side effects, mid- or long-term problems, and social problems. For type of user, marginalised users were chosen as the reference category, as they clearly deviate from the other two subsamples. For country, Ireland was chosen as the reference category, because respondents from the Irish sample most often reported negative experiences. Life time use of NPS categories were entered as dummy variables (1 = yes, 0 = no). See Table 6. Results indicate that online community NPS users—but not night life users—have less often experienced acute unpleasant side effects from NPS use than marginalised users, that Irish NPS users have experienced more side effects than NPS users from other countries, and that life time use of herbal blends and/or pure synthetic cannabinoids, branded and/or pure stimulants, and dissociative NPS—but not psychedelic or other NPS is associated with unpleasant side effects. These associations between group, country and category of NPS used are independent from each other. Differences between countries, for instance, exist regardless of group of NPS users or life time use of categories of NPS. To illustrate this: among Irish marginalised life time users of stimulant NPS, 97.8% have ever experienced acute unpleasant side effects of NPS use, while among Hungarian marginalised life time users of stimulant NPS, this proportion was significantly smaller at 80.5% (chi-square = 7.553, *p* = .006). Thus, while the large differences between countries mainly reflect the varying distribution of user groups, there are also significant differences in the same user group by country. As for mid- or long-term problems, these are associated with being a marginalised user, living in Ireland, Hungary or Poland, and life time use of herbal blends and/or pure synthetic cannabinoids, branded and/or pure stimulants, and dissociative NPS. Social problems are associated with being a marginalised user, living in Ireland or Hungary, and life time use of herbal blends and/or pure synthetic cannabinoids, branded and/or pure stimulants, dissociative NPS, and other NPS.

**Table 6:** Regression analysis of side effects and problems from NPS use across groups and lifetime use of NPS

	Acute unpleasant side effects			Mid- or long-term problems			Social problems		
	B	S.E.	p	B	S.E.	p	B	S.E.	p
Marginalised (reference category)									
Night life	− .309	.220	.159	− .951	.187	.000	− 1.076	.192	.000
Online community	− .683	.207	.001	− .986	.172	.000	− 1.139	.174	.000
Ireland (reference category)									
Germany	− 2.115	.619	.001	− 1.118	.329	.001	− 1.632	.349	.000
Hungary	− 1.702	.622	.006	− .095	.330	.773	− .510	.348	.142
The Netherlands	− 3.048	.616	.000	− 1.190	.323	.000	− 2.485	.348	.000
Poland	− 1.634	.616	.008	− .485	.319	.129	− .820	.338	.015
Portugal	− 3.181	.630	.000	− 1.803	.369	.000	− 2.125	.390	.000
Life time use of herbal blends and/or pure synthetic cannabinoids	.556	.099	.000	.430	.106	.000	.596	.121	.000
Life time use of branded and/or pure stimulants	.460	.115	.000	.728	.126	.000	.799	.135	.000
Life time use of psychedelics	.094	.095	.325	− .087	.101	.389	.104	.119	.383
Life time use of dissociatives	.473	.118	.000	.337	.118	.004	.265	.129	.039
Life time use of other NPS	− .030	.101	.763	.181	.102	.077	.377	.115	.001
Constant	2.453	.607	.000	.222	.316	.484	.275	.336	.414
Nagelkerke R^2^	.236			.184			.309		

## Discussion

This study was undertaken within the remit of a transnational and interdisciplinary EU funded research project undertaken by researchers from different scientific disciplines in six EU-countries: Germany, Hungary, Ireland, the Netherlands, Poland and Portugal. Limitations of the prevalence and other data centre on the varied composition of country samples. Key findings centre on the particular trends visible in NPS use across countries, and the three identified NPS user groups (marginalised, nightlife and online). Most notable and similar to other research (Measham et al. 2011a; Van Hout 2016) was that the concept of NPS was not always clear at consumer level, with some users not clear on what NPS category they had used, and were unaware of the active ingredients. Additional limitations centred on the general nature of questions of negative experiences when using NPS which could have biased associations when multiple NPS categories were used within poly drug using episodes. We also recognise that respondents may have been recollecting negative experiences and may have since ceased using that particular NPS.

Proportions of marginalised users were larger in samples of Hungary and Ireland, while the Portuguese sample consisted mainly of night life users and German and Dutch respondents mainly of online community users. Poland was the only country with a more even distribution of user groups. However, regardless of sample composition, several clear differences in NPS use were evident. In terms of profile, demographic trends are similar to previous monitoring of NPS (Europol-EMCDDA 2010; Carhart-Harris et al. 2011; Measham et al. 2011b; Werse and Morgenstern 2012; Eurobarometer 2014; Johnston et al. 2015; Martinotti et al. 2015; Global Drug Survey 2016). Recent NPS users in all countries are predominantly male (less than one third female).

In general, recent users of all categories of NPS are mainly young adults aged 18–24 years. The online community sample was the youngest with the majority falling within the 18–24 age group. We know that the internet and social media fuels the marketing, popularity and sale of NPS (Orsolini et al. 2016; Lavorgna 2016) with younger drug users active on online websites (Móró and Rácz 2013; Rosino and Linders 2015; Soussan and Kjellgren 2014, 2016; Van Hout 2014a, b; Van Buskirk et al. 2016; Hearne and Van Hout 2016; Bilgrei 2016; Tzanetakis et al. 2016; Van Hout and Hearne 2015, 2017). Night life users were somewhat older: between 18 and 35 years (average 25.7 years). Most night life users reside in large towns, but online community users often live in small and medium-sized towns. Levels of education are high among both night life and online community users. Marginalised users of NPS were older than the other two samples (average 33.5 years), and predominantly found in large towns, have had little education and were often unemployed.

Use of ‘*herbal blends*’ and ‘*synthetic cannabinoids obtained pure*’ was most prevalent in Germany, Poland and Hungary, and least prevalent in the Netherlands. In contrast, use of ‘*branded stimulants*’ and ‘*stimulants/empathogens/nootropics obtained pure*’ was most prevalent in the Netherlands, and least popular in Portugal. Diffusion of both categories has been reported elsewhere (Arfken et al. 2014). The Portuguese and German samples are distinguished by high levels of ‘*psychedelic*’ NPS use, and ‘*dissociative*’ and ‘*other*’ NPS were highest in Germany and lowest in Ireland. In terms of most popular NPS used in these countries, all three categories of NPS users reported majority use of stimulants. This reflects trend data which reports on high demand for synthetic stimulants in Europe due to displacement from traditional drugs such as amphetamine, MDMA and cocaine. With regard to the number of different substances detected in previous years, synthetic cathinones are the second largest group of NPS (EMCDDA 2015). Similar trends are described on contemporary drug fora on the Surface Web (Van Hout 2014b; Van Hout and Hearne 2015; Sande 2016; Hearne and Van Hout 2016).

Similar to Hearne and Van Hout (2016), marginalised NPS users also differed from night life and online community users in terms of reporting a higher prevalence of use of herbal blends and/or synthetic cannabinoids and stimulants, a lower prevalence of psychedelic NPS use, and had less awareness of the active synthetic ingredients in herbal blends. Online community users distinguish from both marginalised and night life users by relatively high levels of benzodiazepine class NPS. This has also been observed in online NPS users active on the Darknet (Van Hout and Hearne 2017) and as popular trend in online pharmacies (Littlejohn et al. 2005). There was no difference between NPS user categories in terms of dissociative NPS. Route of administration differed between groups, with marginalised users using commonly via injection, a mode that was used by barely anyone from the other groups. A history of intravenous drug use was relatively prevalent among users of ‘*herbal blends*’, ‘*synthetic cannabinoids obtained pure*’ and ‘*dissociatives*’ NPS.

Dissociatives and synthetic cannabinoids are the substances that are mostly associated to health and social problems. It is important to note that poly use of NPS compounds (sequential and concurrent) and NPS with conventional illicit drugs is common and can exacerbate such acute effects (Van Hout and Hearne 2017). That said, the reported acute side effects support those described in the literature (Stepens et al. 2008; Auwärter et al. 2009; Every-Palmer 2010; Hadlock et al. 2011; Maan and D’Souza 2011; Dargan et al. 2010, Vearrier and Osterhoudt 2010; Lusthof et al. 2011; Kasick et al. 2012; Grandy 2012; Seely et al. 2012; Gunderson et al. 2012; Tung et al. 2012; Werse and Morgenstern 2012; King and Nutt 2014; Soussan and Kjellgren 2015, 2016; Van Hout 2014b; Abdulrahim et al. 2015; Van Hout 2016; Bassir et al. 2016; Van Hout and Hearne 2016, 2017) and include experiences of increased heart rate/palpitation, dizziness, anxiety/horror trips and headaches as the most common symptoms across all three categories. Life time use of herbal blends and/or pure synthetic cannabinoids, branded and/or pure stimulants, and dissociative NPS, but not psychedelic or other NPS is associated with such unpleasant side effects. Online community NPS users, but not night life users, reported less often experiences of acute unpleasant side effects from NPS use than marginalised users. Generally, the relatively high rates of acute side effects experienced by users with experiences in synthetic cannabis use are in line with prior observations that users of such substances report more, and quite divergent, negative side effects than users of other NPS (Werse and Egger 2016), as well as toxicological research that assesses such substances as much more risky than cannabis with regard to overdose and other physical symptoms (Moosmann and Auwärter 2016). Further comparative research is needed to assess whether symptoms experienced by users of particular NPS categories differ from the symptoms experienced after the use of common illicit drugs of the same class of substances.

Marginalised users not only reported substantially more acute and unpleasant side effects than other respondents, but reported particularly much more mid- and long-term mental and physical problems (addiction withdrawals and craving, weight loss, depression and paranoid disorders) and social problems (conflict with partner or family, problems with housing and legal problems) as a consequence of NPS use. These results could be expected, given the risk behaviours associated with injecting and dependent drug use, as well as the precarious social situation of drug users who are marginalised by definition. Mid- and long-term problems are associated with being a marginalised user, living in Ireland, Hungary or Poland, and life time use of herbal blends and/or pure synthetic cannabinoids, branded and/or pure stimulants, and dissociative NPS. Addiction withdrawals and craving were reported by almost half of marginalised users along with weight loss, depression and paranoid disorders. Currently, there is a limited demand for specialist NPS treatment in Europe, despite some Member States identifying a need for such clinical and treatment services (EMCDDA 2016). Social problems are associated with being a marginalised user, living in Ireland or Hungary, and life time use of herbal blends and/or pure synthetic cannabinoids, branded and/or pure stimulants, dissociative NPS, and other NPS. Low proportions of online and nightlife users reported social or legal problems in terms of conflict at school or university, with partners or family, or with housing.

## Conclusion

The study is illustrative of the need for continued surveillance and monitoring of NPS trends across Europe. The development of country-specific information resources, awareness raising and prevention initiatives particularly around the contents of popular NPS is warranted. Appropriate clinical responses to acute side effects and mid- to long-term problems associated with NPS use across the three NPS user groups are needed, along with enhanced social service supports for NPS user groups experiencing associated problems. Country-specific initiatives in response to user trends should include the regular training of health and social care professionals, health and social service needs assessments, and targeted service responses to reduce incurred harms.
